# Suppression of Hepatitis C Virus Core Protein by Short Hairpin RNA Expression Vectors in the Core Protein Expression HUH-7 Cells

**DOI:** 10.1080/15257770701503787

**Published:** 2007-12-10

**Authors:** Hitoshi Suzuki, Hiroyasu Kaneko, Nobushige Tamai, Kunitada Shimotohno, Naoko Miyano-Kurosaki, Hiroshi Takaku

**Affiliations:** Department of Life and Environmental Sciences, Chiba Institute of Technology, Chiba, Japan; Department of Viral Oncology, Institute for Virus Research, Kyoto University, Kyoto, Japan; Department of Life and Environmental Sciences, Chiba Institute of Technology, Chiba, Japan, High Technology Research Center, Chiba Institute of Technology, Chiba, Japan

**Keywords:** RNAi, Short hairpin RNAs (shRNAs), hepatitis C virus (HCV) core protein, Huh-7 cells, GFP, DsRed

## Abstract

Short hairpin RNAs (shRNAs) efficiently inhibit gene expression by RNA interference. Here, we report the efficient inhibition by DNA-based vector-derived shRNAs of core protein expression in Huh-7 cells. The shRNAs were designed to target the core region of the hepatitis C virus (HCV) genome. The core region is the most conserved region in the HCV genome, making it an ideal target for shRNAs. We identified an effective site on the core region for suppression of the HCV core protein. The HCV core protein in core protein-expressing Huh-7 cells was downregulated by core protein-shRNA expression vectors (core-shRNA-452, 479, and 503). Our results support the feasibility of using shRNA-based gene therapy to inhibit HCV core protein production.

## INTRODUCTION

Hepatitis C virus (HCV) is one of the main causes of liver-related morbidity and mortality. The virus establishes a persistent liver infection, leading to the development of chronic hepatitis, liver cirrhosis, and hepatocellular carcinomas. However, a highly effective anti-HCV drug has yet to be developed, due in part to the lack of detailed information about the HCV life cycle.

While aiming to develop an alternative treatment to interferons and focusing on gene therapy, we adopted a method of using RNA interference (RNAi) based on short hairpin RNA (shRNA), which is expected to yield good treatment effects as a therapeutic gene. As the target sequence, we selected the HCV core protein gene.^[^1–4^]^ Conservation of the core protein sequence is extremely high among HCV genes. Because HCV functions as an mRNA and has a single strand genomic RNA, it is expected that cleavage of the core protein mRNA will inhibit nuclear transport and virus duplication.^[^5^]^ We designed three shRNAs against the following regions of the HCV core protein sequence: 452 to 472 nt, 479 to 499 nt, and 503 to 523 nt. We designed shRNA expression vectors targeting the core protein site and compared their inhibitory effects.

## MATERIALS AND METHODS

### Plasmid Constructs

We designed DNA-based vectors expressing shRNA. Sense and antisense strands of shRNA oligonucleotides were synthesized and then annealed at 95°C for 3 min, followed by slow cooling in phosphate buffered saline (pH 7.4, containing 50 mM NaCl). These oligonucleotides contained the loop CCACACC sequence. The annealed oligonucleotides were designed to have KpnI and BamHI ends and these ends were inserted into the pU6 vector, which is based on pSV2-neo. A Pollll-type promoter was used for shRNA expression. The constructed plasmids, pU6-core-shRNA-452, pU6-core-shRNA-479, or pU6-core-shRNA-503, were used in the experiments. The plasmids pU6-core-shRNA-452, pU6-core-shRNA-479, and pU6-core-shRNA-503 were named to correspond with their respective targets in the HCV core protein regions (452–472 nt, 479–499 nt, and 503–523 nt, respectively). Scrambled shRNA (control) cloned into the same vector was used as a negative control in all experiments. The inhibitory effects of the three shRNAs were compared using a cell line produced by transducing the core protein expression vector (pEF1-core) into Huh-7 cells.

### Fluorescence Microscopy

To determine the intracellular localization of the transfected shRNA expression vectors, Huh-7 cells (2 × 10^4^ cells) were singly transfected or cotransfected with pGFP-core (nuclear exporting vector), pDsRed-PA28γ, and core-shRNA expression (pU6 plasmid) vectors, using the FuGENE 6 reagent according to the manufacturer's protocol, and were cultured for 48 h at37°C in a 5% carbon dioxide atomosphere. Fluorescent cells were examined with a confocal microscope (Zeiss LSM5 PASCAL; Carl Zeiss, Jena, Germany) at excitation wavelengths of 543 nm and 488 nm, using a 40× objective. Images were acquired at a 512 × 512 resolution.

### Chemiluminescent Enzyme Immunoassay

We determined the efficacy of HCV core protein inhibition with the pEF1-core protein vector (0.5 μg) transfected into Huh-7 cells. pEF1-core protein vector (0.5 μg) and pU6-core shRNA (2 μg) were co-transfected into Huh-7 cells using the FuGENE 6 transfection reagent. After 48 h, intracellular HCV core protein was measured using an HCV core protein antigen chemiluminescent enzyme immunoassay (CLEIA) assay. HCV core protein antigen levels were determined using a fully automated CLEIA system according to the manufacturer's procedure.

### RT-PCR

To determine the efficacy of pU6-shRNA-mediated gene silencing, the vectors were transiently transfected into HCV core protein-expressing Huh7 cells (4 × 10^4^) using the FuGENE 6 transfection reagent according to the manufacturer's protocol.

The mRNA content was assessed by reverse transcriptase-polymerase chain reaction (RT-PCR) at 48 h post-transfection and related to the amount produced in the absence of pU6-shRNA.

## RESULTS AND DISCUSSION

**FIGURE 1:**
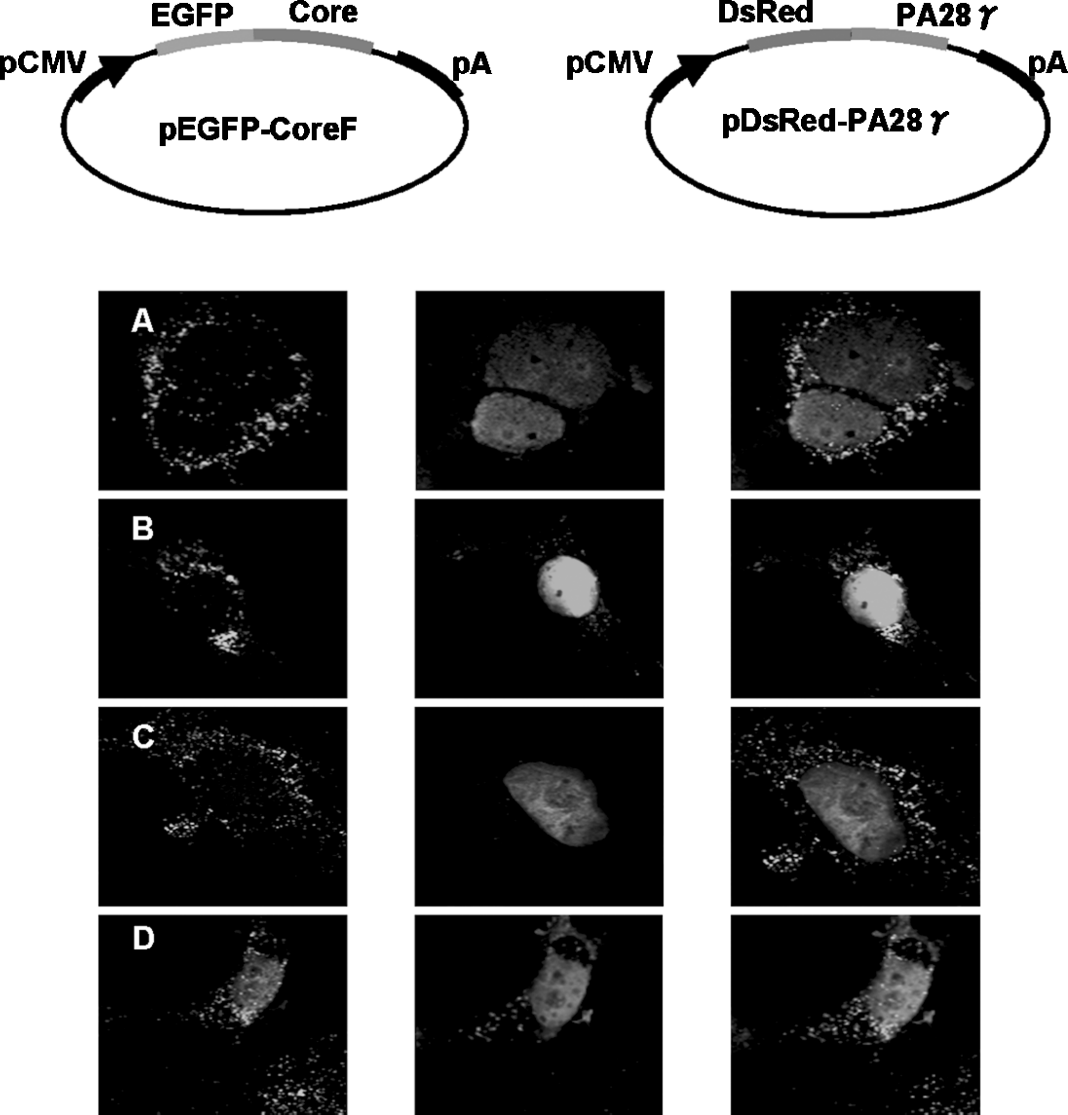
Intracellular localization of triple-transfected expression vectors in Huh-7 cells. Plate-A: Triple transfection of pU6-shRNA-452, pGFP-core (nuclear exporting vector), and pDsRed-PA28γ vectors into Huh-7 cells. Cleavage of the GFP-core protein mRNA, which is the target of shRNA-452, generated expression only in the cytosol in the GFP image, while both the DsRed and merged images show nuclear expression. Plate-B: Triple transfection of the pU6-shRNA-479, pGFP-core, and pDsRed-PA28γ vectors into Huh-7 cells. Cleavage of GFP-core protein mRNA, which is the target of shRNA-479, generated expression only in the cytosol in the GFP image, while both the DsRed and the merged images show nuclear expression. Plate-C: In a similar fashion, pU6-shRNA-503, together with pDsRed-PA28γ and pGFP-core mRNA, expressed exhibited cytoplasmic localization in the GFP image. Plate-D: The scrambled version of all of the pU6-shRNAs was transfected with the pDsRed-PA28γ and pGFP-core vectors. Nuclear expression is shown in the GFP, DsRed, and merged images.

The expression of shRNAs targeting specific portions of the HCV core protein in core protein-expressing Huh-7 cells is a critical factor for effective silencing. We confirmed the inhibitory effect of the shRNA in the cells using fluorescence microscopy. The pGFP-core, pDsRed-PA28γ, and shRNA expression vectors were simultaneously inserted into Huh-7 cells and the localization of the GFP-core was assessed by fluorescence microscopy after a 48 h culture. The core proteins were localized in the cytoplasm of the core protein-shRNA (core-shRNA-452, 479, and 503)-expressing Huh-7 cells. In the control cells (shRNA-scramble), the core proteins were localized in the nucleus (Figure 1). This result indicates that the core protein-shRNA inhibited the expression of the core proteins.

**FIGURE 2:**
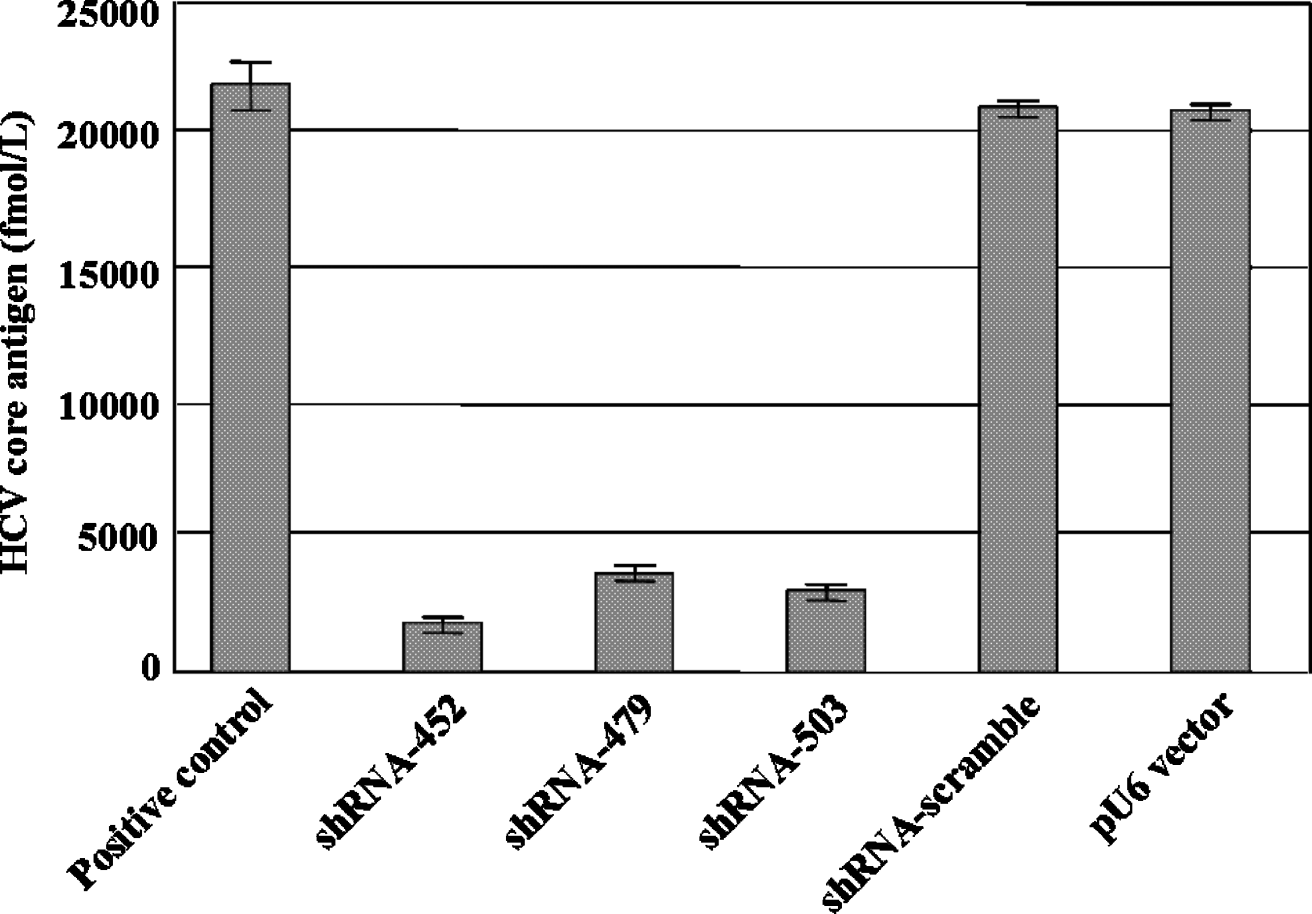
Inhibition of HCV replication by core protein-shRNAs in Huh-7 cells. The pEF1-core vector (0.5 μg) and the pU6-core-shRNAs (2 μg) were co-transfected into Huh-7 cells (4 × 10^4^). After 48 h, the amount of intracellular HCV core protein was measured using the HCV core protein antigen CLEIA assay.

**FIGURE 3:**
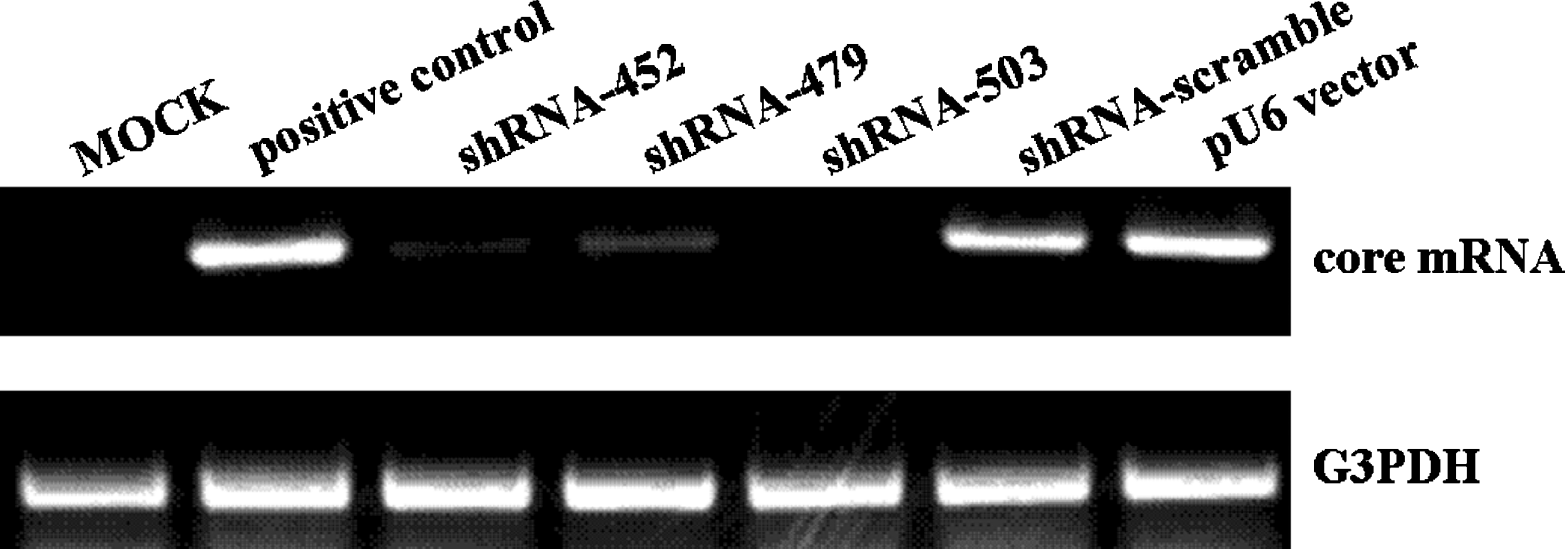
Inhibition analysis of expressed HCV core protein mRNA by shRNA expression vectors using an RT-PCR assay. The pEF1-core vector (0.5 μg) and pU6-core-shRNAs (2 μg) were co-transfected into Huh-7 cells (4 × 10^4^). After 48 h, total RNAs were extracted and subjected to RT-PCR.

The inhibition of core protein production was then evaluated by CLEIA, to determine whether the core proteins actually existed. The pEF1-core and each of the shRNA expression vectors were co-transfected into Huh7 cells, and the cells were lysed and analyzed 48 h later. The results revealed that core-shRNA-452, core-shRNA-479, and core-shRNA-503 inhibited the expression of the HCV core protein (Figure 2). The inhibition mediated by the core-shRNA-452 was much stronger than that of either core-shRNA-479 or 503. In addition, the intracellular HCV core protein mRNA levels were determined by RT-PCR. The pEF1-core and each of the shRNA expression vectors were co-transfected into Huh-7 cells. The presence of the HCV core protein mRNA in the Huh-7 cells was examined by RT-PCR 48 h post-transfection. The shRNAs suppressed the expression of the core protein mRNA (Figure 3). Our study demonstrates that direct sequence-specific degradation mediated by shRNA expression in the core protein-expressing Huh-7 cells downregulates HCV core protein. Based on these results, shRNA expression vectors targeted to the core region might be useful as anti-HCV agents.

The results from this study support the potential use of shRNA expression vectors as a gene therapy approach to HCV replication, which might prove to be a valuable means of treating HCV infections.
